# Reliability and validity of the Turkish version of the elder abuse suspicion index in community-dwelling older adults

**DOI:** 10.55730/1300-0144.5600

**Published:** 2023-01-04

**Authors:** Eray Serdar YURDAKUL, Betül Gülsüm YAVUZ VEİZİ, Candeniz AVCI, Hatice Tuğba YAZIR, Elif AVANER, Mehmet İlkin NAHARCI, Oktay SARI

**Affiliations:** 1 Department of Medical History and Bioethics, Gülhane Faculty of Medicine, University of Health Sciences, Ankara, Turkey; 2 Division of Geriatrics, University of Health Sciences, Gülhane Faculty of Medicine & Gülhane Training and Research Hospital, Ankara, Turkey; 3 Department of Family Medicine, Gülhane Faculty of Medicine & Gülhane Training and Research Hospital, University of Health Sciences, Ankara, Turkey

**Keywords:** Older adults, elder abuse, validity and reliability, Toronto declaration, vulnerable populations

## Abstract

**Background/aim:**

Elder abuse is among the most important ethical issue during the management of older population. The elder abuse suspicion index (EASI) was developed for evaluating abuse in older adults. We aimed to assess the reliability and validity of the Turkish version EASI-Türkiye (TR) among older adults.

**Material and methods:**

This study included 89 community-dwelling older adults. The EASI-TR and other scales, including Hwalek-Sengstock Elder Abuse Screening Test-Türkiye (HS/EAST-TR), YGDS, Yesavage Geriatric Depression Scale (YGDS), Instrumental Activities of Daily Living (IADL), and Activities of Daily Living (ADL) were administered to all participants. Internal consistency and external validity were assessed.

**Results:**

EASI-TR revealed an excellent test-retest reliability and acceptable level of internal consistency (Cronbach’s α = 0.711). The item-total correlations ranged between 0.296 and 0.701, except for the second item. This test showed significant correlations with the HS/EAST-TR and IADL (p < 0.05), demonstrating good external validity.

**Conclusion:**

The EASI-TR appears to have acceptable reliability and validity in screening for abuse in older adults. This tool may recognize cases that require additional evaluation in managing of ethical issues.

## 1. Introduction

Elder abuse, which leads to numerous ethical challenges, can be defined as direct action, inaction, or negligence that causes harm or increases its risk in accordance with the Toronto Declaration on Global Prevention of Maltreatment of Older People [1]. This behavior is usually committed by people close to the older person, such as caregivers or relatives. It can involve physical, sexual, psychological, or economic exploitation [1]. Elder abuse usually includes simultaneous exposure to more than one type of abuse [1, 2]. Unfortunately, this abuse is a community health concern. A metaanalysis found a combined prevalence rate of 15.7% for this issue [3]. A systematic review conducted in our country reported that 13.3%–28.5% of the elderly were abused [4]. Importantly, elder abuse is often unrecognized, although it may increase comorbidity burden and death risk at advanced age [1].

There are many difficulties in detecting elder abuse, which are almost unnoticed in many parts of the world [5]. The absence of notifications may be caused by factors such as isolation of the elderly from society, the level of perception of abuse as a problem, and the attitudes of the family toward this issue [5, 6]. In addition, it is difficult to reach consistent findings on the prevalence of abuse due to differences in the definitions of legal regulations on abuse and the inadequacy or lack of studies on this issue in developing or underdeveloped countries [5, 7]. On the other hand, inadequate awareness of physicians and other health professionals dealing effectively with this ethical issue, who will make the notification by making the diagnosis of abuse, is also an important factor in these rates [7].

The inadequacy of descriptive measurement tools is an important problem in the diagnosis of abuse [8]. Limited easy-to-apply scales are available to evaluate and diagnose elder abuse in outpatient settings [9].

The EASI is a brief screening test used to assess elder abuse in large samples [10–12]. It is also a simple tool for use in research and clinical practice on abuse of older individuals [10, 13]. The EASI has translations into several languages in different cultures and ethnic groups [13, 14], yet it has not been evaluated for Turkish speaking individuals. Thus, we aimed to investigate the reliability and validity of the Turkish version of EASI (EASI-TR) for clinical use in older adults.

## 2. Materials and methods

### 2.1. Design

Eighty-nine community-dwelling older subjects (≥65 years) who lived independently were prospectively included in the study. Mild cognitive impairment or dementia, active psychiatric distress (having a diagnosis of depression and receiving antidepressant treatment), and delirium within the last 3 months were the exclusion criteria.

Participant’s evaluations were made face-to-face when they were alone in the outpatient clinic, with guaranteed confidentiality. Participants with severe hearing or visual impairments that could reduce the evaluation performance of the clinical tests and those with missing data were also excluded.

Sociodemographic characteristics, comorbidities (diabetes, hypertension, stroke, and coronary artery disease), and hearing aid and eyeglass use data were collected for each participant. The study was approved by the local ethics committee and the written informed consent was obtained from all participants (2022/1648-463).

A participant/item ratio of at least 5:1 was utilized to calculate the minimum number of participants required [15, 16]. EASI contains six questions; therefore, we used the formula 6 (items) × 5 (at least number per item) = 30 when reckoning the number of participants required.

### 2.2. Elder abuse suspicion index (EASI)

This test is a short screening measure, helping physicians assess the suspicion of elder abuse [17]. It consists of six short questions applicable to older adults without cognitive impairments. Answering the YES to one or more questions 2,3,4,5, and 6 may suggest mistreatment. It has a total summed score of 5 (minimum score = 0 and maximum score = 5) and takes 2–5 min to complete. The doctor questions the first five queries and the participant answers them as yes or no. Next, the doctor answers the last query in the light of his/her observations about the participant. The first question of the EASI is not included in the scoring because it evaluates distress and is not sign of suspected maltreatment [17].

### 2.3. Assessment tools

#### 1. Elder abuse

Hwalek–Sengstock Elder Abuse Screening Test (H-S/EAST)

This questionnaire is a short screening measure, identifying the suspected signs of elder abuse [18]. This test includes 15 questions in three categories: direct harassment, vulnerability features, and suspected abusive conditions [18]. The score on this scale ranges from 0 to 15. Higher scores suggest greater risk, with ≥3 points indicating older adult abuse [18].

#### 1. Physical function

The Lawton Instrumental Activities of Daily Living (IADL) instrument is used to evaluate independent households, including shopping for groceries, using transportation, using the telephone, preparing meal, housework, laundry, taking medication, and handling finances [a summed score ranging from 0 (low function) to 8 (high function)] [19].

The Katz Activities of Daily Living (ADL) instrument is used to evaluate self-care tasks, including feeding, bathing, dressing, toileting, continence, and transferring [a summed score ranging from 0 (low function) to 6 (high function)] [20].

#### 2. Mood assessment

The short form of the Yesavage Geriatric Depression Scale (YGDS) is used to screen for depressed mood in older persons. Total scores range from 0 to 15 (higher scores indicating more depressive symptoms) [21].

### 2.4. Translation and adaptation

This process included three phases: (I) forward-translation (made by two translators), (II) back-translation (made by two different translators), and (III) cross-cultural adaptation, consistent with the method developed by Beaton [22]. Researchers and translators reviewed all six items on the scale for the need to make the necessary changes. We then transferred the latest version to the researchers of the EASI to check for agreement or changes. Lastly, this tool was tested in a small group of subjects with normal cognition before it was used in this study.

### 2.5. Statistical analysis

We performed analyses using IBM SPSS version 24.0. The Kolmogorov–Smirnov test was used to assess the normality distribution of the data. We reckoned internal consistency using Cronbach’s α, considering the questions within the EASI-TR. A Cronbach’s α value higher than ≥0.70 is considered acceptable [23]. Item-total correlations were calculated, with correlations above 0.20 considered acceptable. The intraclass correlation coefficients (ICC) were calculated by measuring test–retest reliability (temporal stability) [24]. We evaluated criterion-related validity by comparing the EASI-TR with the HS/EAST-TR, which is the gold standard scale to assess elder abuse, using Spearman’s r correlation coefficients. Convergent validity was evaluated by comparing the EASI-TR with the corresponding scales, including IADL, ADL, and YGDS, using Spearman’s r correlation coefficients [25]. A value of p < 0.05 in the analyses was considered significant.

## 3. Results

The mean age was 75.3 ± 7.0 years, and 56.4% were women. Almost two-thirds of the sample had an education level of 8 years or less. More than half of them were married and living with spouses. While the proportion of eyeglass use was high (%83.1), only 8 participants required a hearing aid (%9.0). The most common chronic disease was hypertension (80.9%), followed by diabetes mellitus (40.4%) and ischemic heart disease (13.5%) (Table 1).

The mean HS/EAST-TR and the EASI-TR scores were 6.0 ± 1.3 and 1.1 ± 0.9. The proportion of suspected abuse, based on the index test, was 27%. In addition, 4.5% of the participants had depressive symptoms (YGDS score ≥ 6). All participants were functionally independent, with mean IADL score of 7.0 ± 1.5 and ADL score of 5.8 ± 0.5. All characteristics of the study sample are presented (Table 1).

The overall Cronbach’s α coefficient was 0.711, ranging from 0.602 to 0.731 of questions, indicating that the internal consistency was acceptable. Test–retest reliability was excellent [ICC value = 0.978 (0.970–0.986)]. The “alpha if item deleted” analysis revealed that deleting questions second and fourth increased the overall Cronbach’s α score. The item-total correlations were positive and ranged from 0.296 to 0.701, except for the second question (Q2 = 0.001) (Table 2).

*Convergent validity*. The total test score was negatively related to the IADL score (r = −0.445, p < 0.001) (Table 3). EASI-TR questions, including Q1 and Q6, were also negatively related to the IADL score (r = −0.421, p < 0.001; r = −0.259, p = 0.024, respectively). However, the total EASI-TR score did not correlate with the ADL and YGDS (r = −0.132, p = 0.255; r = 0.087, p = 0.456, respectively) (Table 3).

*Criterion-related validity*. The total test score was positively related to the HS/EAST-TR score (r = 0.324, p = 0.002) (Table 3). The EASI-TR questions, including Q3, Q4, Q5, and Q6, were also positively correlated with the HS/EAST-TR scores (r = 0.304, p = 0.004; r = 0.242, p = 0.023; r = 0.298, p = 0.005; and r = 0.333, p = 0.001, respectively).

## 4. Discussion

In this study, we found that the EASI-TR has acceptable validity and internal reliability among community-dwelling older Turkish adults. Considering that elder abuse is poorly known and unreported, the EASI-TR can be used as an instrument to suspect abuse, which is a growing problem in care. In addition, almost a quarter of the participants experienced some form of mistreatment. Therefore, our study can make significant contributions to recognizing of this condition in health services and the development of public policies to protect vulnerable adults.

Cronbach’s α coefficient of EASI-TR indicated an acceptable level of internal consistency, proving that the items were correlated with each other. Accordingly, our findings suggest that EASI-TR can be safely used for detecting abuse in the older Turkish population. Although the EASI is a simple and quick screening measure for elder abuse [26] and has been translated into many languages, we could not find results reported in the literature for direct comparison with our results. Moreover, in a pilot study evaluating the adaptation of the EASI to Ireland, this test was shown to be routinely used by more than one professional in different settings [27]. However, in a project involving seven participating countries, WHO found that not all questions in the EASI are appropriate for all cultures and cannot be asked in all settings, and therefore does not yet recommend the universal use of the questionnaire [26]. As a result, further studies on reliability are needed for older adults with larger samples in different countries.

Remarkably, we found that the level of internal consistency of EASI-TR would increase if the second and fourth questions were removed from the questionnaire. The “corrected item-total correlation” values of these questions were very close to the lowest limit for Q4 and negatively correlated for Q2. The sensitivity of these questions was also found to be low in the original EASI study [17]. These questions may point to a specific culprit by directly asking about negligence and financial abuse. The abused older adult may not answer the questions correctly so as not to disrupt family solidarity, out of a sense of humiliation and shame so as not to punish the perpetrator, or for fear of losing their current care [28]. However, this may also occur in other situations, such as when the older person normalizes elder abuse and sees himself or herself as a burden to the caregiver [29]. Although the EASI form is considered a good and simple tool that covers all major categories of abuse, additional efforts are necessary to enhance community-based older adults’ confidence for mistreatment screening instruments to be used appropriately [26, 29].

Consistent with the findings of previous studies on elder abuse and functional status, this tool presented a strong convergent validity with the IADL test for the total score [30–32]. The stress of caring for a functionally limited person may lead to abuse [31]. On the other hand, we did not find any association of EASI-TR with ADL in our study. The reason could be that our study included patients with an MMSE score >24 who were relatively independent in functional tasks and who could come to the outpatient controls alone or with their relatives. Notably, using this test in individuals with poor cognitive and functional status could adversely affect the accuracy of results. In line with this, patients with dementia or functional disability were not enrolled in the original EASI study [17]. Furthermore, conflicting reports exist regarding the association between mistreatment and depression [33–36]. Depression is both a trigger and result of elder abuse [29, 33]. Nevertheless, in our study, no significant convergent validity was found between the total YGDS score and EASI-TR. This could be due to underreporting of depressive symptoms in older adults or the cross-sectional design, which may not enable full psychiatric evaluation [37, 38].

In addition to these sufficient degrees of convergent validity, testing the criterion-related validity of a screening instrument necessitates comparison with a gold standard scale. Although there are challenges in establishing such a standard method for abuse in older adults [39–42], the H-S/EAST instrument, previously validated and reliable in the Turkish population, can be used as the most appropriate comparative test for detecting elder abuse [43]. In our study, the correlation of the EASI-TR with the H-S/EAST provided a satisfactory level of criterion-related validity. However, further research on validity is required to verify our findings.

As a secondary outcome, we found that 27% of the older adults may have met elder abuse. In line with our findings, a pilot study in which the WHO assessed the use and cultural appropriateness of EASI involving seven countries reported that elder abuse was 30% [26]. However, three studies conducted in different societies reported that the proportion of elder abuse varied from 3.2% to 18.4% [27, 44–47]. These differences in the ratios of elder abuse may stem from differences in standard definitions and screening tools, and the difficulty in detecting abuse at advanced ages, which is influenced by coexisting medical and social problems and internal issues such as denial [28, 48, 49].

Some limitations of this study should be considered when interpreting its results. Firstly, the EASI-TR is only appropriate for older adults with intact cognitive capacity. However, this does not provide an opportunity to study patients with mental health problems. Secondly, this study was done in an outpatient clinic. Therefore, studies involving multiple centers are needed by confirming and disseminating these results to other regions of Türkiye. Finally, there is no established gold standard instrument for comparison with the EASI-TR. A lot of special instruments related to elder abuse have been developed; however, only the H-S/ EAST test was eligible in the Turkish population [43].

As abuse in older adults is an increasing issue worldwide, healthcare providers require new assessment tools. Our results emphasize that the EASI-TR is a valid and reliable instrument that can be used in outpatient monitoring without wasting time during screening for mistreatment. However, further studies are required by evaluating EASI-TR at different settings. This study also provides a basis for our future ethical research regarding vulnerable groups, such as older adults, children, and prison inmates.

Raising societal awareness of abuse in older adults, identifying aggrieved persons, and intervening early are considerable points. For this reason, public health professionals need to develop different strategies for social services to ensure ethical behavior among clinicians, who report incidents when they suspect elder abuse and are sensitive to the victim.

## Figures and Tables

**Table 1:** Characteristics of the study population.

Variables		Total (N = 89)
**Sociodemographics**		
Age categories (years), n (%)		
	65–74	46 (51.7)
	75+	43 (48.3)
Age (years), mean ± SD		75.3 ± 7.0
Sex (female), n (%)		52 (58.4)
Education	≤8 years	61 (68.5)
Marital status		
	Married	51 (57.3)
	Other a	38 (42.7)
Living status		
	Spouse	52 (58.4)
	Other b	37 (41.6)
Hearing aid (yes), n %		8 (9.0)
Eyeglass (yes), n (%)		74 (83.1)
**Comorbidities (yes), n (%)**		
Hypertension		72 (80.9)
Diabetes mellitus		36 (40.4)
Ischemic heart disease		12 (13.5)
Stroke		2 (2.2)
**Elder abuse status**		
HS/EAST-TR (0–15), mean ± SD		6.0 ± 1.3
EASI-TR (0–5), mean ± SD		1.1 ± 0.9
**Depression status**		
YGDS (0–15), median (min–max)		0 (0–11)
**Physical status**		
IADL (0–8), mean ± SD		7.0 ± 1.5
ADL (0–6), mean ± SD		5.8 ± 0.5

HS/EAST-TR, Hwalek-Sengstock Elder Abuse Screening Test-Türkiye; EASI-TR, Elder Abuse Suspicion Index-Türkiye; YGDS, Yesavage Geriatric Depression Scale-15; IADL, Instrumental Activities of Daily Living; ADL, Activities of Daily Living.

a Other; unmarried, widowed, or divorced.

b Other; alone or relative.

Results are mean ± SD or percentage or median (min–max).

**Table 2:** The psychometric properties of the EASI-TR.

Questions	Internal consistency		Corrected item-total correlations
	Cronbach’s alpha	Cronbach’s alpha if item deleted	
Q1		0.662	0.486
Q2		0.731	<0.200
Q3		0.677	0.488
Q4		0.714	0.296
Q5		0.695	0.474
Q6		0.602	0.701
Total	0.711		

EASI-TR, Elder Abuse Suspicion Index-Türkiye

**Table 3:** Correlations of the EASI-TR with elder abuse, depression and functional assessment tools.

Tools	Spearman’s correlation coefficient (r)	*p*
*Elder abuse*		
HS/EAST-TR	0.463	**<0.001**
*Depression status*		
YGDS	0.087	0.456
*Physical status*		
IADL	− 0.445	**<0.001**
ADL	− 0.132	0.255

HS/EAST-TR, Hwalek-Sengstock Elder Abuse Screening Test-Türkiye; EASI-TR, Elder Abuse Suspicion Index-Türkiye; YGDS, Yesavage Geriatric Depression Scale-15; IADL, Instrumental Activities of Daily Living; ADL, Activities of Daily Living.

Values given in bold indicate statistically significant results (p < 0.05).
